# Triple metachronous primary cancer of uterus, colon, and breast cancer: A case report and review of the literature

**DOI:** 10.1097/MD.0000000000021764

**Published:** 2020-08-21

**Authors:** Guanqiao Li, Jia Yao, Tangna Wu, Yaxiong Chen, Zhenping Wang, Yiming Wang, Fen Wang, Rui Zhong, Shiping Yang

**Affiliations:** a Department of Breast Surgery; b Department of Radiation Oncology; c Department of Ultrasound, Hainan General Hospital (Hainan Affiliated Hospital of Hainan Medical University), Haikou, Hainan, China.

**Keywords:** breast, colon, triple primary cancer, uterus

## Abstract

**Rationale:**

Triple or more primary malignancies are rare, with only 23 previous cases including breast cancer reported in the English language studies between January 1990 and December 2019.

**Patient concerns:**

The patient was a 67-year-old woman with a mass in her right breast. She had a previous history of uterine and colon cancer. Both ultrasonography and mammography revealed a Breast Imaging Reporting and Data System (BI-RADS) category 3 breast lesion, in which proliferative nodules are more likely. Given her previous history of 2 malignancies, her doctors strongly recommended a biopsy.

**Diagnosis and interventions:**

The biopsy pathology suggested intraductal breast cancer. Mastectomy and sentinel lymph node biopsy were performed. The postoperative pathological diagnosis was invasive ductal carcinoma, grade II, stage I. The sample was positive for estrogen receptor and progesterone receptor and negative for cerbB-2. No radiotherapy or chemotherapy was administered except for endocrine therapy. A follow-up at 19 months showed no breast recurrence or distant metastases.

**Outcomes:**

No recurrence or distant metastasis occurred within the 19-month, 11-year, and 20-year follow-ups for breast, colon, and uterine cancers, respectively.

**Lessons:**

To our knowledge, this is the first review of triple or more primary malignancies including breast cancer. These malignancies occur predominantly in older female patients. The most prevalent tumors of triple or more primary malignancies including breast cancer occur in the colon, uterus, and lung. A favorable prognosis is associated with early-stage malignancies.

## 1 Introduction

Among multiple primary malignancies (MPM), 2 cancers are common, whereas triple or more primary malignancies are rare. According to the time of diagnosis, MPM can be classified as synchronous or metachronous. The reported incidence of MPM in cancer patients ranges from 0.52% to 11.7%.^[1]^ However, there is limited literature regarding treatment and prognosis for MPM.

We reviewed cases of triple or more primary malignancies including breast cancer reported in English in the past 30 years (January 1990–December 2019) and found reports of 23 previous cases of triple or more primary malignancies including breast cancer.

Here, we report a rare case of metachronous triple primary malignant tumors of the uterus, colon, and breast. We also summarized the 23 previous cases and the present case (Table 1).^[2–24]^ To our knowledge, this is the first report of triple or more primary malignancies including breast cancer. We received written consent from the patient to publish this case report. An approval from the Institutional review committees was not required because this is a case report.

**Table 1:**
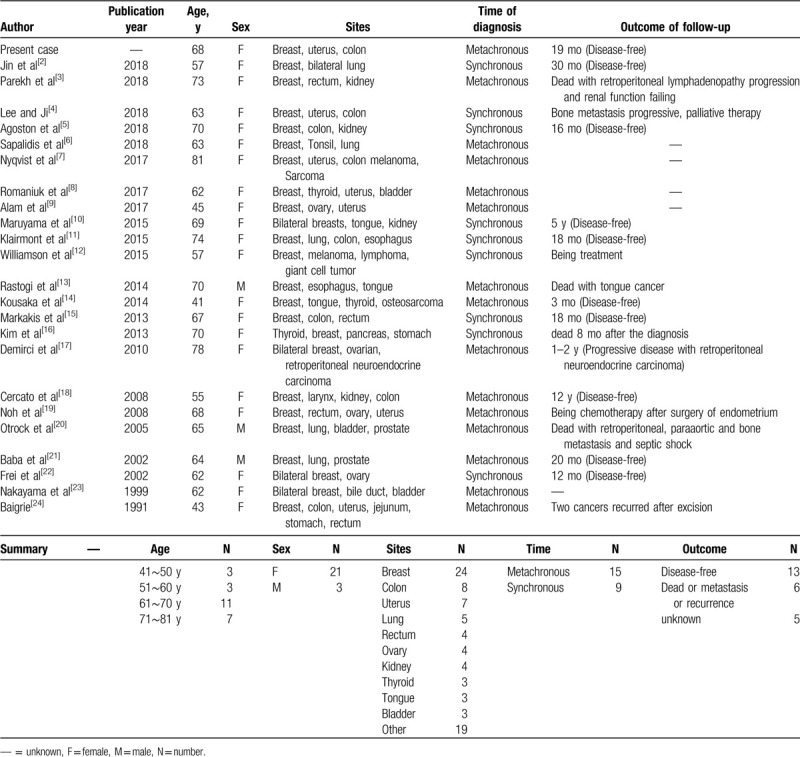
Previous 23 cases and the present case with triple or more primary malignancies including breast cancer.

## 2 Case report

**Figure 1:**
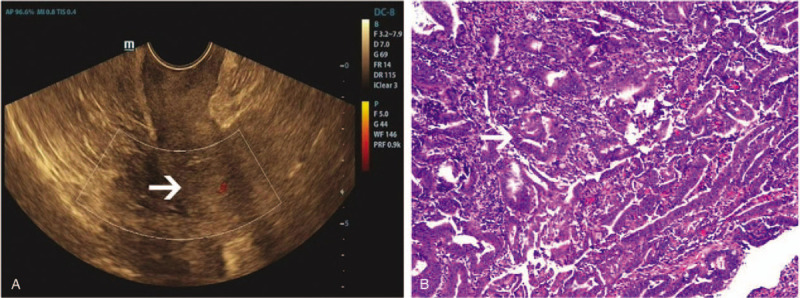
Ultrasound (A) showed a regular, mixed echoic mass (15 mm × 10 mm). HE staining (B) revealed moderately differentiated endometrioid adenocarcinoma (white arrow).

The patient was an older woman who had been diagnosed with 3 different types of cancer in 3 different organs during a 19-year period between 1999 and 2018. In November 1999, the patient visited a gynecology department for irregular vaginal bleeding lasting 1 month. The patient was diagnosed with her first malignancy, endometrial cancer, in 1999 at the age of 48 years. Transvaginal ultrasound revealed a regular, mixed echoic mass (15 mm × 10 mm) at the inferior posterior wall of her uterus (Fig. 1A) diagnostic curetaage was performed.

The endometrium pathologic examination showed endometrial cancer. The patient also underwent a radical hysterectomy and pelvic lymphadenectomy. The histopathological examination specimen (Fig. 1B) revealed moderately differentiated endometrioid adenocarcinoma, with tumor invasion of the muscularis and cervical orifice. The surgical margins were negative and none of the 41 axillary lymph nodes excised were positive for malignancy. The postoperative pathological diagnosis was endometrial cancer, grade II, stage II. She received adjuvant radiotherapy for her pelvic cavity after the operation. No recurrence or metastasis had occurred after 20 years of postoperative follow-up.

**Figure 2:**
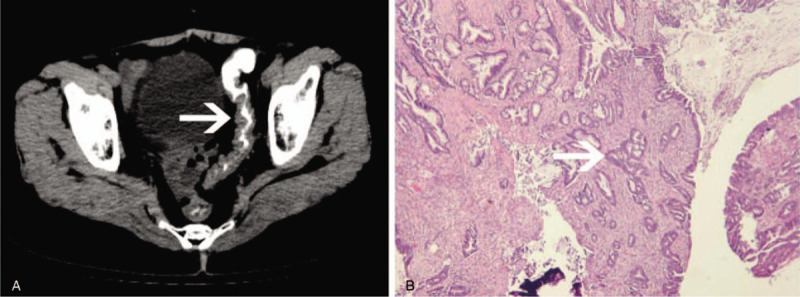
Computed tomography (A) revealed intestinal wall was thickened (white arrow). HE staining (B) revealed ascending colon papillary adenocarcinoma.

In July 2008, the patient experienced recurrent right lower abdominal pain for 2 months. She was diagnosed with her second malignancy, colon cancer, in 2008 at the age of 57 years. Abdominal computed tomography (CT) revealed a thickened intestinal wall in the left lower abdominal sigmoid colon and stenosis of the lumen (Fig. 2A). She underwent fiberoptic endoscopy for the suggested ascending colon cancer (photographs of the fiberoptic endoscopy were lost due to the relocation of the endoscopy center). A pathological biopsy revealed a tubular adenocarcinoma. The patient underwent a right hemicolectomy under general anesthesia on July 13, 2008. Postoperative pathology (Fig. 2B) revealed ascending colon papillary adenocarcinoma and moderately differentiated tubular adenocarcinoma with components of mucinous adenocarcinoma, complicated with deep muscle invasion. The surgical margins were negative and none of the 33 axillary lymph nodes excised were positive for malignancy. The tumor was pathologically categorized as stage I. The patient was treated with traditional chemotherapy comprising 6 cycles of oxaliplatin and 5-fluorouracil. She remained free of locoregional recurrence or distant metastases after 11 years of postoperative follow-up.

**Figure 3:**
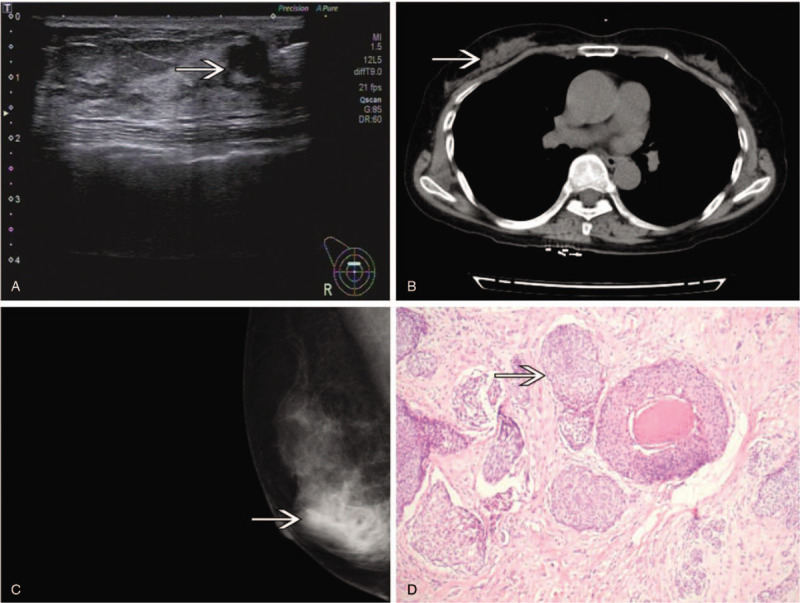
Ultrasound showed a regular, mixed echoic mass (A); CT scan (B) suggested an irregular mass (12 mm × 10 mm) behind the nipple (white arrow); Mammography (C) revealed a nodular high-density lesion (20 mm × 18 mm) with inhomogeneous; (D) revealed that ductal carcinoma in situ of the breast.

In June 2018, the patient sought medical advice for possible breast surgery after finding a mass in her right breast for 2 weeks. She was diagnosed with her third malignancy, breast cancer, in 2018 at the age of 67 years. Breast ultrasound and mammography were performed. The ultrasonography (USG) revealed a regular, mixed echoic mass (8.8 mm × 5.0 mm) at the 12 o’clock region of her right breast (Fig. 3A) categorized as Breast Imaging Reporting and Data System (BI-RADS) category 3, consideration of proliferative nodules. CT revealed an irregular mass (12 mm × 10 mm) behind the nipple of the right breast consistent with the USG findings (Fig. 3B). Mammography (Fig. 3C) revealed a nodular, inhomogeneous, and irregular high-density lesion (20 mm × 18 mm) behind the nipple in the right breast that was categorized as BI-RADS category 3, proliferative nodules are more likely. Although all imaging examinations pointed to a benign nodule, the doctors strongly recommended that the patient undergo a biopsy because of her previous history of multiple cancers. The biopsy pathology suggested intraductal breast cancer. A mastectomy and sentinel lymph node biopsy was performed. Postoperative pathology showed invasive ductal carcinoma of the right breast with a maximum diameter of 1.5 cm, grade II, ductal carcinoma in situ (about 80%) (Fig. 3D), and no sentinel lymph node metastasis (0/2). Immunohistochemistry showed estrogen receptor and progesterone receptor positivity (90% and 3%, respectively). The sample was negative for cerbB-2 but was positive for Ki-67 (5%). No radiotherapy or chemotherapy was administered except for endocrine therapy. A follow-up at 19 months showed no breast recurrence or distant metastases.

## 3 Discussion

Altogether, 23 previous cases of triple or more primary malignancies including breast cancer have been reported in English language studies between January 1990 and December 2019.^[2–24]^ According to the summaries of previous reports^[2–24]^ (Table 1), this type of malignancy occurs predominantly in older female patients, with an average age of 64 years (range 41–81). Our case was 67 years. Fifteen (62.5%) cases were metachronous and nine (37.5%) cases were synchronous. The most prevalent tumor was colon cancer (8 cases), followed by uterus cancer (seven cases) and lung cancer (five cases). The follow-up period of most published cases was relatively short. Eight cases experienced dead/metastasis or recurrence.^[3,4,13,16,17,19,20,24]^ The outcomes of cases were not reported.^[6–9,23]^

The combination of MPM with uterus, colon, and breast cancers has been reported in 1 previous study, in which Lee and Ji reported a case of a 63-year-old woman simultaneously diagnosed with uterine carcinosarcoma, breast cancer, and colon cancer. However, the time of follow-up was very short in that case and the patient had just finished chemotherapy.^[4]^

In these cases, it is important to determine that the second primary cancer is an independent primary cancer rather than a metastasis or recurrence of the first primary cancer as this difference affects staging, treatment, and patient prognosis. In our case, the patient had undergone surgery for endometrium, colon, and breast cancer for which the primary cancers were confirmed by pathology.

There are no guidelines for the diagnosis and treatment of multiple neoplasms; therefore, they are difficult to treat. Yoshino et al^[25]^ reported that “the prognosis of patients with multiple primary cancers can be determined independently by the stage of each cancer.” Bae et al^[26]^ stated that “one of the most important factors when deciding the best treatment modality for patients with multiple primary cancer is the stage of the cancers.” Primary cancers have much better survival rates than metastatic cases.^[27]^ When properly managed, patients’ individual circumstances should be considered, including parameters such as disease grade, extent of neoplastic invasion, patient age, and disease type.^[28]^ The management modalities could include surgical debulking, adjuvant radiotherapy, and adjuvant chemotherapy, as clinically appropriate. More aggressive management modalities may be required for patients with synchronous primary neoplasms, poor histological, higher grade, and advanced stage.

The patient in the present case had been diagnosed with multiple cancers at different times in her life. The primary cancer (endometrial cancer) was diagnosed in December 1999, the second occurrence of cancer (colon cancer) was diagnosed 9 years later in August 2008, and, finally, 10 years after this in 2018, the third cancer (breast cancer) was diagnosed. Due to the long intervals between the cancers, the treatments for all 3 were similar. However, it is curious that the patient received 6 weeks of chemotherapy after an operation for colon cancer and was diagnosed postoperatively as having stage T2N0M0 (stage I) disease. Chemotherapy should not be used in this situation according to National Comprehensive Cancer Network (NCCN) guidelines. We inquired with the consultant who treated this patient; however, he was unable to recall the treatment because it had happened so long ago and had not saved records from that time. The colon and breast cancer were diagnosed relatively early (stage I), whereas the endometrial cancer was stage II at diagnosis.

This patient had a relatively favorable prognosis associated with early-stage malignancies without lymph node metastases (breast, colon adenocarcinoma cancer, and endometrium adenocarcinoma). After the treatment of the first cancer, patients undergo regular follow-up for early identification of the next primary cancer. This patient's second (colon cancer) and third (breast cancer) primary cancers were both stage I. Due to the early diagnosis and treatment, the patient was able to go on to live a long life. The second or third primary malignancies mostly occur in long-term survivors and may be related to environmental, reproductive, genetic, and lifestyle factors (smoking, drinking, and body mass index).

Reports have indicated that 8% of MPM cases are associated with radiotherapy; the remaining cases were correlated with lifestyle behaviors (eg, smoking), and genetic factors.^[28]^ The patient in the present case had no family history or history of smoking or drinking. She received pelvic adjuvant radiotherapy was performed for endometrial cancer in this case. Thus, we cannot rule out whether the colon cancer was related to radiation. Kumar et al^[29]^ evaluated reported the association between radiotherapy and subsequent second cancers in endometrial carcinoma survivors.

In this case, the point of early diagnosis in breast cancer was very noteworthy. Both ultrasound and mammography revealed BI-RADS grade category 3. BI-RADS category 3 lesions have a malignancy rate of >2% at mammography^[30]^ and in most diagnostic ultrasound series.^[31,32]^

Barr et al^[33]^ suggested that “as BI-RADS category 3 lesions have a low malignancy rate (0.8%) and only 0.1% of the cancers had suspicious changes at 6-month follow-up and only one (17%) of six malignancies were node-positive at detection (24-month follow-up), a recommendation of 1-year diagnostic follow-up may be appropriate for BI-RADS category 3 lesions detected at screening US.”

Graf et al also reported that follow-up USG was an acceptable alternative to biopsy for solid masses with benign morphologic features seen in USG owing to the extremely high negative predictive values (99.8%).^[34]^

In the present case, the doctors strongly recommended a biopsy because of the patient's previous history of multiple cancers. She apparently benefited from biopsy and received timely diagnosis and treatment. Therefore, for patients with a history of multiple cancers, the possibility of malignancy should be considered for BI-RADS category 3 lesions and positive puncture biopsy should be performed for definite diagnosis to avoid delayed diagnosis and treatment.

## Acknowledgments

The authors are thankful to the patient for her cooperation. The authors also thank Mr Dean Moody for revising our manuscript for mistakes and grammatical errors.

## Author contributions

Data acquisition: YS, YJ, WF and WY. Data analysis and interpretation: LG, YJ, and ZR. Radiological analysis of ultrasound and CT images: WT, CY, WZ. Manuscript preparation: LG and YS.
